# Negotiating queer identity and cultural meaning through translation in China

**DOI:** 10.3389/fpsyg.2026.1828477

**Published:** 2026-04-22

**Authors:** Guoxi Chai, Xiaogang Jia, Xiaoming Tian, Zeyu Zhang, Haonan Zhen

**Affiliations:** 1 School of Foreign Languages, Yan’an University, Yan’an, Shaanxi, China; 2 School of International Studies, Xi’an University of Finance and Economics, Xi’an, Shaanxi, China; 3 School of Humanities and Foreign Languages, Xi’an University of Posts and Telecommunications, Xi’an, Shaanxi, China

**Keywords:** Bourdieu, China, cultural constraint, identity negotiation, queer translation

## Abstract

**Introduction:**

How individuals negotiate marginalized identities within restrictive sociopolitical environments remains a critical question for understanding the dynamic relationship between culture and the self. This study investigates why and how Chinese professional translators engage with materials related to lesbian, gay, bisexual, transgender, queer, intersex, and other sexual and gender minorities (LGBTQI+) in a sociocultural context where queer identities are frequently marginalized or silenced.

**Methods:**

Conceptualizing translation as a process of cultural and identity negotiation, the study draws on semi-structured interviews with 23 professional translators who have experience translating queer-related literary, academic, or community materials.

**Results:**

Thematic analysis identifies four translator positions that reflect different strategies of engagement with cultural constraints: visibility-seekers, who leverage sociocultural trends for professional or commercial gain; cautious negotiators, who adapt queer content to meet censorship and market norms; insider advocates, queer translators who use their lived experience to affirm cultural identity and empower their community; and allied activists, non-queer translators who engage in ethical solidarity and social justice through translation. These positions reveal how translators mobilize professional resources, personal experience, and ethical commitments while navigating political regulation, cultural stigma, and market expectations.

**Discussion:**

The findings highlight translators as socially situated agents whose practices mediate the circulation of marginalized cultural meanings and demonstrate how identity and cultural expression are co-constructed within structures of ideological constraint.

## Introduction

In contemporary societies marked by globalization and cultural circulation, individuals increasingly negotiate their identities within complex sociopolitical environments where cultural meanings are unevenly distributed and contested. One important yet understudied arena of such negotiation is translation, which not only transmits linguistic content but also mediates cultural meanings and shapes the visibility of marginalized identities. In China, censorship and ideological restrictions on queer content substantially shape the practice of translators working with related materials. This study investigates how professional translators negotiate such sociopolitical constraints when translating queer materials, a term used by the participants in this study to describe literary, academic, and community materials concerning queer topics.

The broader Chinese sociopolitical climate imposes stringent limitations on the overt representation of queer identities, compelling translators across media to negotiate visibility through strategic and often subtle forms of mediation. For instance, in audiovisual translation, the 2018 television adaptation *Chen Qing Ling* (The Untamed), based on the Chinese novel *Mo Dao Zu Shi*, exemplifies how ideological pressures shape translational choices. In this adaptation, the translator adapters reframed the protagonists’ romantic gay relationship into a platonic ‘socialist brotherhood’ (Ng and Li, 2020), a strategy that preserved queer subtext while conforming to state-sanctioned narratives. Such cases illustrate how translation practices become embedded within broader cultural and political processes that regulate the expression of sexuality and identity in public discourse.

Similar tensions pervade the translation of Anglophone television series, films, and books into Chinese. For instance, mainland Chinese streaming platforms, such as Youku and iQiyi, have directly censored queer content through translation and editing, including the removal of same sex kisses from *Friends* (an American television sitcom) and the deletion of queer subplots from *Modern Family* (an American television sitcom) (Chen, 2022). At the same time, translators must navigate not only institutional censorship but also cultural sensitivity and audience expectations. The mainland Chinese translation of *Call Me by Your Name—*a 2017 coming-of-age romantic drama film based on André Aciman’s novel, omits all erotic scenes and reframes the novel’s sensuality through lyrical euphemism, exemplified by the rendering of ‘restless longing’ as the more abstract and restrained term ‘desire.’ Likewise, in the Chinese translation of *The Velvet Rage*, a self-help book by clinical psychologist Alan Downs, ‘gay’ was translated as ‘comrade,’ a term laden with local political and historical resonance, to sustain queer legibility within Chinese discourse (de Van Werff, 2008). These examples demonstrate how translation mediates between global cultural narratives and local ideological constraints, shaping the forms through which queer identities become visible or intelligible within specific sociocultural contexts.

State censorship, online content removals, and the shutdown of queer support organizations further marginalize these communities (Gao et al., 2023; Zhao, 2020). This marginalization directly affects translation practices, forcing translators to dilute, euphemize, or omit queer content in order to meet publication requirements or protect themselves from reputational risk (Jin and Ye, 2023). While some translators conform to these pressures through self-censorship, others, particularly grassroots queer fan subbing communities, mobilize translation and paratextual practices to create queer spaces and pursue non-confrontational forms of advocacy and resistance under censorship (Guo and Evans, 2020; Song, 2024). In this sense, translation can be understood as a socially situated practice through which individuals negotiate the circulation of marginalized cultural meanings while responding to institutional and cultural constraints.

The term ‘queer,’ as adopted by the participants in this study, carries both symbolic and operational significance. Drawing on Bao’s (2021b) insight, it functions simultaneously as a neutral euphemism that minimizes ideological scrutiny and as a community-recognized marker circulating on Chinese social media platforms. This dual function reflects professional translators’ efforts to maintain cultural authenticity while maneuvering within politically sensitive environments. Against this backdrop, this study argues that translators’ engagements with queer materials are shaped by uneven configurations of habitus and capital, resulting in divergent orientations ranging from pragmatic accommodation and strategic ambiguity to cautious advocacy. Moreover, translators’ positions across overlapping translation fields, including commercial publishing, audiovisual media, and grassroots circulation, further condition both the degree of risk they can assume and the strategies they deploy. These dynamics reveal translation as a socially embedded practice in which ideological constraint, professional calculation, and personal disposition intersect, raising two major research questions:

RQ1: How do censorship and sociocultural pressures in China shape translators’ engagement with queer materials?

RQ2: How do translators navigate these pressures through habitus and capital-based strategies in translation and circulation?

## Literature review

Over the past two decades, sexuality related scholarship, including research on LGBTQI+ health, identities, and social attitudes, has expanded across disciplines such as public health, biomedicine, and the social sciences (Gkinopoulos et al., 2024; Lo Moro et al., 2024; Ventriglio et al., 2022). Within this broader landscape, queer translation studies have emerged as an interdisciplinary area of inquiry that examines how sexuality, language, and power intersect in processes of cross-cultural circulation. Recent special issues foregrounding queer translation and queer popular culture reflect growing scholarly interest in this field; however, this body of research has largely been shaped by academic agendas and publishing infrastructures in the Global North, particularly in the United States and Europe (Baldo et al., 2021; Brown, 2012; Keenaghan, 2023). As a result, the sociopolitical conditions under which queer materials are translated and circulated in non-liberal or authoritarian contexts remain comparatively underexamined.

Existing global scholarship on queer translation has predominantly focused on textual strategies and linguistic negotiation, exploring how translators render queer narratives, bodies, and identities across languages and cultural contexts (Baer, 2020; Baer and Kaindl, 2017; Epstein and Gillett, 2017; Gramling and Dutta, 2016). More recent studies have extended this line of inquiry to audiovisual media, popular culture, and activist settings, highlighting translation as a site for negotiating normativity, visibility, and accessibility (Attig, 2021; Baldo et al., 2021; Martínez Pleguezuelos, 2021). In parallel, a growing number of scholars have begun to address translator identity and agency in the production of queer discourse (Asma, 2022; Felix, 2014; Pan and Yi, 2017). Nevertheless, relatively little attention has been paid to how structural pressures, such as censorship regimes and dominant sociocultural norms, shape translators’ modes of participation, professional positioning, and risk assessment, particularly in politically restrictive environments.

In the Chinese context, research on sexuality and queer-related topics is subject to stringent ideological filtering, which constrains both academic publication and public visibility (Perry, 2020; Wei, 2020). Irvine (2014) underscores the role of academic publishing in shaping research agendas, noting that the marginalization of sexuality studies contributes to the limited circulation and recognition of such work. For translators, these conditions translate into layered constraints arising from state censorship, publishing house gatekeeping, and culturally entrenched taboos surrounding sexual content. These constraints shape how translators negotiate lexical choices and representational strategies in interaction with editors and other institutional actors within the publishing field (Cao, 2022). Studies of censored translations produced in the People’s Republic of China, such as analyses of Chinese versions of *The Catcher in the Rye*, demonstrate the use of deletion, attenuation, and modulation when references to homosexuality or sexual content are deemed unacceptable to publishers, regulators, or mainstream audiences (Guo, 2022). These challenges are further intensified by the absence of standardized Chinese terminology for many queer concepts, which contributes to interpretive ambiguity and reinforces marginalization through lexical invisibility (Bao, 2021a).

Within this constrained publishing ecology, translators occupy complex and often precarious positions shaped by the dual demands of commercial viability and ideological compliance (Jin and Ye, 2023). These pressures require translators to balance professional ethics, personal dispositions, and political risk when engaging with queer materials. While existing studies document censorship effects at textual or institutional levels, there remains a notable lack of empirical research into how translators themselves perceive, negotiate, and respond to these pressures in their everyday practices, as well as how such responses vary across different translation fields and career trajectories. Much of the current literature prioritizes textual outcomes over translators’ lived experiences and sociological positioning. Addressing this gap, this study adopts Bourdieu’s theoretical framework, particularly the concepts of field, habitus, and capital, to examine how censorship and sociocultural pressures shape translators’ engagement with queer materials in China (RQ1), and how translators mobilize differentiated forms of capital to navigate these constraints across multiple translation contexts (RQ2).

## Theoretical consideration

This study draws on Bourdieu’s (1987) concepts of *field, habitus,* and *capital* to examine and explain how translators of queer materials in China position themselves within structurally constrained environments. These concepts provide analytical tools for understanding how translators’ practices are shaped by the interaction between sociocultural pressures, professional conditions, and individual dispositions (Bourdieu, 1987). Although Bourdieu’s framework has been widely applied in gender-related research on masculine domination, academic achievement, and workplace dynamics (e.g., Edgerton et al., 2014; Fowler, 2003; Polić and Holy, 2021), its use in studies of queer translation remains limited.

Focusing on professional translators working with queer materials, this study examines how habitus, formed through personal trajectories, professional training, and cultural experience, informs translators’ positioning and orientations toward risk. It also considers how different forms of capital, including cultural, symbolic, social, and economic capital, are mobilized to negotiate legitimacy and agency within fields shaped by censorship, heteronormativity, and market logics. Rather than treating translation decisions as purely individual choices, the analysis approaches translators’ strategies as position-takings emerging from the interplay between internalized dispositions and external field conditions.

*Field* is understood as a structured social space characterized by unequal access to resources, recognition, and authority (Bourdieu, 1987). In the context of queer translation in China, translators operate across overlapping fields, including commercial publishing, audiovisual media, and grassroots circulation, each imposing distinct constraints and risk configurations. Translators’ positioning within and across these fields’ conditions both the degree of risk they can assume and the strategies they adopt, ranging from strategic accommodation to selective advocacy.

*Habitus* functions as an embodied system of dispositions that shapes how translators perceive risk, interpret constraints, and evaluate possible courses of action (Bourdieu, 1987). This perspective accounts for the differentiated positioning observed in this study, including pragmatic compliance, cautious neutrality, and identity-informed advocacy. Translators embedded in queer communities may draw on a habitus attuned to representational ethics and affective resonance, while others rely on professional or empathetic dispositions to balance personal commitments with institutional expectations.

*Capital* provides the resources through which translators enact their positioning within the field. Cultural capital, such as familiarity with queer discourse and genre conventions, symbolic capital in the form of professional recognition, social capital rooted in community networks, and economic capital linked to market opportunities, are differentially activated depending on translators’ orientations and risk assessments. For example, some translators accumulate symbolic and economic capital through popular queer genres, while others mobilize social and cultural capital to sustain queer visibility under restrictive conditions.

This conceptual framework is further informed by queer theory, which critiques heteronormativity as a regulatory force embedded in language, culture, and translation practices (Butler, 1990; Warner, 1993). In China’s family-centered and morally conservative context (Li, 2019), queer materials are frequently subject to censorship or normative adjustment (Harvey, 2000). Translators thus occupy a mediating position in which their field-based positioning and capital deployment shape how queer meanings are circulated, constrained, or sustained.

## Materials and methods

This study examines what motivates Chinese professional translators to engage with queer materials and how they position themselves within fields structured by sociopolitical regulation, cultural norms, and professional expectations. Adopting a qualitative research design, the study employs semi-structured interviews to capture translators’ situated accounts of risk negotiation, field positioning, and capital mobilization in their engagement with queer translation.

The research team consists of three university faculty members from different institutions, all holding professional translation qualifications, conducting research in translation studies, and supervising master’s students, as well as two postgraduate students currently pursuing a master’s degree in translation. Throughout the research process, we remained attentive to how our academic training, institutional locations, and prior assumptions might shape data generation, interpretation, and representation. Rather than centering researcher sexual identity categories, we approached this issue through methodological reflexivity, with particular attention to our interpretive responsibilities in representing participants’ lived experiences. To address these asymmetries, we adopted ethical and reflexive research practices throughout, including informed consent, participant review of interview transcripts, anonymization of identifying details, and sensitivity to contextual language use. We therefore approached the study as a collaborative and ethically attentive inquiry, aiming to represent participants’ positioning, experiences, and strategies with care, accuracy, and analytical caution.

Given the political and cultural sensitivity surrounding queer issues in China, participant recruitment relied on snowball sampling. Initial participants were contacted through professional translation networks known for engagement with marginalized topics, and subsequent participants were referred by translators willing to discuss the risks and constraints associated with queer translation. Recruitment initially focused on three tier-one cities, Beijing, Shanghai, and Guangzhou, selected for their concentration of translation programs, relatively high exposure to global cultural flows, and their role as sites where cultural innovation and regulatory control intersect. As recruitment progressed, the sample expanded through professional networks and snowball sampling, resulting in a geographically diverse group of participants. In total, 23 professional translators from 17 cities across China’s five major geographic regions (Eastern, Western, Southern, Northern, and Central) were recruited for the study (see Table 1).

**Table 1:** Interviewee bio-data and interview thematic distribution.

Pseudonym	Sex	Sexuality	Region	Years of translation experience	Working language	Key themes
Lin	Male	Heterosexual	Northern	7	CE	Commercial gain, queer fandom trends
Song	Male	Heterosexual	Eastern	9	CE	Audience preferences, CP fandom
Hua	Female	Not Stated	Southern	8	EC	Royalties, market demand
Min	Female	Heterosexual	Central	11	CE/EC	Scholarly neutrality, content selection
Jiang	Male	Not Stated	Western	14	EC	Linguistic compromise, euphemism
Xiu	Female	Lesbian	Northern	6	CE	Insider cultural capital, community authenticity
Qing	Male	Gay	Eastern	5	CE/EC	Identity affirmation, intellectual exploration
Bo	Male	Heterosexual	Southern	10	EC	Equal knowledge access, anti-discrimination
Ying	Female	Heterosexual	Central	7	CE/EC	Feminist solidarity, affective fidelity
Tao	Male	Heterosexual	Western	8	CE	Strategic visibility, professional prestige
Jia	Female	Heterosexual	Northern	6	EC	Cautious engagement, risk avoidance
Kun	Male	Gay	Eastern	9	CE/EC	Community advocacy, resistance to stigma
Lan	Female	Bisexual	Southern	7	CE	Intersectional identity, nuanced translation
Qiang	Male	Heterosexual	Central	12	EC	Academic curiosity, cultural enrichment
Mei	Female	Heterosexual	Western	5	CE/EC	Compassionate allyship, emotional resonance
Feng	Male	Heterosexual	Northern	10	CE	Market alignment, popular queer genres
Shu	Female	Not Stated	Eastern	8	EC	Euphemistic translation, censorship compliance
Yang	Male	Gay	Southern	6	CE/EC	Insider perspective, community empowerment
Ling	Female	Heterosexual	Central	9	CE	Ethical responsibility, social justice
Xiao	Male	Heterosexual	Western	7	EC	Accessibility, challenging heteronormativity
Dan	Female	Lesbian	Northern	8	CE	Cultural sensitivity, queer narrative preservation
Peng	Male	Heterosexual	Eastern	11	CE/EC	Scholarly objectivity, non-confrontational representation
Yue	Female	Heterosexual	Southern	6	EC	Allyship, amplifying marginalized voices

CE = Chinese to English; EC = English to Chinese. Sexuality marked as “Not Stated” indicates that participants declined to disclose this information. To protect participants’ identities, and because the number of professional translators in some cities is very small, specific city names are not reported; only regional locations are provided.

All participants had a minimum of 5 years of professional translation experience and at least 1 year of sustained engagement with queer materials, including literary works, academic texts, audiovisual media, digital content, or activist materials. This range reflects the differentiated structure of the translation field and translators’ varied positioning across genres and circulation contexts. Six participants had also attended queer-related conferences or community events, indicating differing levels of cultural, social, and symbolic capital accumulation.

Semi-structured interviews were conducted to elicit reflective accounts of how translators’ habitus shapes their orientations toward queer materials and their assessments of professional and sociopolitical risk. Interview questions focused on participants’ personal trajectories, professional positioning, experiences with censorship, and the ways they mobilize different forms of capital to achieve visibility, legitimacy, or cultural advocacy. Participants were also invited to reflect on how institutional constraints influenced their translation strategies and circulation choices. To protect participants, pseudonyms were used throughout, and identifying information such as institutional affiliations and translated titles was anonymized.

During the interviews, participants frequently used region-specific dialectal expressions to articulate their translational attitudes, strategies, and experiences, which not only reflect the geographic diversity of the queer translation field but also carry nuanced cultural and contextual meanings. These dialectal expressions, their regional origins, English glosses, and corresponding interview contexts are documented in Table 2 to preserve the cultural specificity of participants’ individual accounts and expressions while ensuring academic clarity for readers unfamiliar with Chinese regional dialects.

**Table 2:** Dialectal expressions, regional origins, and interview contexts.

**Dialectal expression**	**Region**	**English gloss**	**Corresponding interview extract context**
骑一波热度	Northern	Ride the wave of popularity	Visibility-seekers describing engagement with queer fandom trends
出圈	Eastern	Gain widespread attention	Highlighting professional visibility from queer translation
实打实的收益	Southern	Tangible benefits	Economic capital from queer translation
不沾边	Central	Have no connection to	Disavowing ideological intent in academic translation
绕着弯子译	Western	Translate in a roundabout way	Linguistic compromise strategies for censorship compliance
懂行	Northern	Understand the ins and outs	Insider cultural capital of queer translators
有那味儿	Eastern	Capture the essence	Authenticity of insider translations
接地气	Northern	Relatable to local contexts	Regional community validation of translations
打抱不平	Southern	Defend against injustice	Allied activists’ motivation for anti-discrimination
搭把手	Western	Lend a hand	Compassionate allyship in translation

All interviews were conducted in Chinese. Interviews were recorded when consent was granted and transcribed verbatim to preserve linguistic and affective nuance. Participants were invited to review transcripts to confirm accuracy and clarify meaning, a procedure consistent with Bourdieu’s (2004) emphasis on reflexivity and relational knowledge production. Data analysis followed Braun and Clarke’s (2021) six-phase thematic analysis framework, adapted through a Bourdieusian perspective to foreground the interaction between field constraints and individual positioning. Coding proceeded iteratively in three stages: open coding to identify recurrent patterns, axial coding to organize these patterns around the analytical categories of field, habitus, and capital, and selective coding to develop themes that captured how translators navigate constraint, risk, and agency in queer translation. Particular attention was paid to recurring meanings, shared experiences, and patterned forms of self-description across participants’ accounts, all of which were interpreted within the thematic analysis process.

Themes such as professional visibility, strategic compromise, ethical allyship, and identity-informed advocacy emerged from the analysis, reflecting translators’ differentiated positioning within a constrained field. Across participants’ accounts, recurring patterns of experience and self-description were used to support the interpretation of these themes. Through these themes, the study illuminates how translators negotiate risk, mobilize capital, and enact professional agency while mediating queer meanings under sociopolitical and institutional pressure.

## Findings

The four themes presented below were developed through thematic analysis of recurrent patterns in the interview data, including repeated accounts, evaluative expressions, and forms of self-positioning used by participants to describe their translational practices. These recurring patterns clustered around four broad orientations toward queer translation: strategic visibility-seeking, cautious negotiation, insider advocacy, and allied activism.

### Strategic translation: negotiating popularity, profit, and pride in translation

Six participants approached queer translation as a strategic practice embedded in market demand and emerging cultural trends, with their accounts consistently oriented toward the accumulation of economic and symbolic capital. As visibility-seekers, they use their cultural knowledge of youth fandoms, online trends, and local audience preferences to strategically position themselves in the commercial translation field. Unlike translators motivated by advocacy, their engagement with queer content is conditional on its market potential, and they actively avoid topics deemed too politically sensitive or niche to attract broad readership, strictly adhering to commercial publishing’s profit-driven logic in both material selection and translational style.

Lin openly framed queer content as a pathway to enhanced professional visibility: *“Translating niche queer content lets me ‘ride the wave of popularity’, a way better than slaving over ordinary texts. Frankly, I’m here to boost my reputation, not just showcase talent.”* He further elaborated on his strategic selection of materials, revealing a clear market-oriented screening mechanism: *“I stick to lighthearted danmei (gay) novels, not the heavy academic stuff or activist texts. Those sell fast on Amazon and get me more client inquiries from publishing houses.”* His account highlights a strong emphasis on personal gain and market opportunity, suggesting an orientation shaped more by career advancement than by ideological commitment to queer issues. His case demonstrates one way in which market-oriented translators may convert familiarity with fandom culture into symbolic visibility and professional opportunity.

Song linked his choices to widespread audience enthusiasm for queer-themed popular culture: *“Whether in the Yangtze River Delta or beyond, young people are obsessed with CP fandom and danmei. Translating this content wins over readers, and that’s what builds my professional standing.”* He noted how he dynamically adapts his translational style to fit regional audience tastes, a key strategy to maximize commercial appeal: *“Readers in Shanghai and Hangzhou love lyrical descriptions of relationships, so I soften the language to fit their preferences, which makes the books ‘gain widespread attention.”* Song’s account reflects an acute awareness of regional cultural trends, treating queer-themed content as a reliable resource for expanding readership and professional influence. His case demonstrates one way in which market-oriented translators may convert familiarity with fandom culture and audience segmentation into symbolic visibility and professional opportunity.

Hua centered material rewards as her primary and most direct motivation for engaging with queer translation: *“Readers in the Pearl River Delta are willing to pay for queer texts, my royalties from these translations let me buy a small apartment. The ‘tangible benefits’ speak for themselves.”* She added specific details about her commercial promotion strategy, which cleverly evades potential censorship while broadening market reach: *“I partner with online bookstores to promote my translations as ‘trendy youth reads’ instead of ‘queer literature’; that way, it avoids censorship red flags and attracts more mainstream buyers.”* This example illustrates how, in some cases, commercial positioning can depoliticize queer content by framing it as lifestyle- or youth-oriented consumption.

Similar market-oriented patterns also appear in the accounts of Tao, Feng, and Peng, all of whom describe queer translation less as cultural or political commitment than as a route to recognition, branding, or stable professional demand. Tao, for instance, treated queer topics as a fast track to academic and professional recognition: *“Popular queer topics get way more buzz than regular content. I do not care about the politics; I care about getting recognized fast.”* Feng similarly framed queer content as a tool for personal brand building, while Peng emphasized its value for professional credibility and funding applications. Across these accounts, queer visibility becomes meaningful primarily insofar as it can be transformed into reputation, opportunity, or economic return.

Thematic analysis across this group suggests a market-driven habitus that often shapes translational decisions. A recurring pattern in participants’ accounts is the prioritization of reputation, professional standing, royalties, and visibility, while queer identity, community needs, and the broader social significance of queer content may remain peripheral in some cases. Their strategies can be understood as converting familiarity with fandom culture and regional audience preferences into professional status and economic benefits, suggesting how the commercial translation field may, in certain contexts, commodify marginalized identities. Rather than simply reflecting individual preference, these accounts point to a potential field logic in which queer visibility becomes valuable insofar as it can be monetized, branded, or made professionally advantageous.

### Safe distance: scholarly engagement and strategic compromise in translation

Five participants adopted the stance of cautious negotiators, engaging with queer materials primarily out of intellectual curiosity and a desire to introduce global queer culture to Chinese audiences, while employing textual and interpretive compromises to avoid censorship and social stigma. Min insisted on strict scholarly detachment as the core principle of her queer translation practice: *“I translate queer texts for their cultural research value, not for activism. Whether my work changes public opinion is not my concern. I’m simply sticking to my role as a translator and have no personal connection to the queer community.”* She also practiced deliberate selective avoidance of potentially controversial content to minimize personal and professional risk: *“I will not touch books that link homosexuality to disease. I do not want readers grilling me and getting me into trouble.”* She elaborated on her specific lexical choices in academic translation, which prioritize neutrality over precision: *“When translating academic articles, I use ‘same-sex attraction’ instead of ‘queer’ or ‘gay’; it’s more neutral and less likely to trigger censorship from journal editors or publishing houses.”* Her account exemplifies a form of self-protective academic neutrality in which terminological precision is subordinated to institutional safety.

Jiang described regionally common strategies of indirect formulations that balance publishing compliance with the preservation of queer meaning: *“Translating queer scenes needs to be ‘in a roundabout way’. ‘They love each other’ becomes ‘they are as close as brothers’, ‘spouses’ becomes ‘bosom friends.’ Publishers approve, and the community gets the subtext; it’s a win-win.”* He defended such linguistic adjustments as an unavoidable occupational realism for queer translators: *“This is not about my beliefs; it’s about keeping my job as a translator of queer materials.”* He shared a concrete example from his audiovisual translation work that exemplifies this compromise: *“In a subtitle project, I changed a same-sex kiss scene description to ‘a moment of deep connection.”* Jiang’s examples illustrate one way in which euphemism can serve not merely as a translational preference but as a practical survival strategy within a censored field.

Qiang, also from Central China, echoed Min’s scholarly orientation but framed his engagement with queer materials through the lenses of academic curiosity and cultural enrichment. As a heterosexual male translator with 12 years of experience in English-to-Chinese translation, he emphasized the intellectual value of queer texts while maintaining a cautious distance from their political dimensions*: “I translate queer literature because it enriches my understanding of global cultural diversity. These texts provide unique perspectives on human experience and broaden my intellectual horizons as a translator.”* He described his approach to sensitive content as one of principled neutrality, selecting materials based on their literary merit rather than social or political significance*: “I focus on queer works that have garnered critical acclaim or literary awards: texts that possess inherent cultural capital. This provides me with a defensible position: I am translating recognized literature, not advocating for a cause.”* Similar to Min, he proactively avoided translations that might invite unwanted attention*: “I steer clear of contemporary queer activist texts or works with an explicit political agenda. My interest lies in the aesthetic and philosophical dimensions of queer writing, not its role in social movements.”* His account reflects a pattern of cautious scholarly engagement shaped by institutional norms and the desire to accumulate cultural capital while minimizing professional risk.

Jia emphasized maintaining a deliberate emotional and professional distance from the queer content she translates, framing caution as her guiding principle: *“I engage with queer content but keep a safe distance. I do not take on projects that are too explicit or politically sensitive. Better to be cautious than regret it.”* She explained the rigorous vetting process she applies to potential queer translation clients and projects to avoid risk: *“I ask for detailed synopses before accepting any work. If the content focuses on queer rights protests or confrontations with authorities, I decline immediately. I only stick to literary works with subtle, implicit queer themes.”* Shu relied on strategic vagueness as her primary tool for navigating censorship, a practice she has refined through years of experience: *“I describe queer relationships as ‘close bonds’ or ‘special friendships’. It’s vague enough to pass publisher and censor review but clear enough for readers in the know to recognize the queer subtext.”* She added that this cautious approach has yielded practical professional benefits: *“My translations have never been rejected by publishers, and I’ve built a reputation as a ‘reliable’ translator for sensitive and niche topics.”*

Thematic analysis shows that this group consistently frames its work through hedging language, neutral framing, and deliberate euphemism to reduce ideological friction and avoid censorship. A recurring theme across these accounts is the preference for professionally sustainable forms of engagement over explicit representational affirmation. They draw on what can be interpreted as cultural capital, derived from intimate awareness of local censorship norms, to navigate translation choices and audience expectations. Their cautious positioning illustrates what Inghilleri (2005) terms ‘nuanced complicity’: strategic compliance with structural constraints that allows for the continued, albeit limited and muted, representation of queer themes in Chinese public discourse. By prioritizing neutrality and strategic vagueness in their translation and framing, they successfully navigate the tension between intellectual curiosity and institutional risk, but their choices also significantly limit the transformative and affirming potential of their translations for queer audiences. What appears as neutrality in these accounts is therefore not politically empty, but a constrained strategy shaped by censorship, professional precarity, and the need to remain institutionally legible.

### Insider advocacy: cultural authenticity and community affirmation in queer translation

Six participants who identified as queer positioned themselves as insider advocates, drawing on their lived queer experiences to make translational choices and engage in community advocacy. Their accounts repeatedly emphasize emotional resonance, insider queer vocabulary, and explicit references to the specific needs of regional queer communities, reflecting an identity-based habitus and a strong, deliberate investment in queer social and cultural capital. Unlike visibility-seekers or cautious negotiators, they prioritize the authenticity, affirmation, and needs of queer audiences above all else, even when this commitment means facing greater professional or social risk, such as publisher pushback or lost projects.

Xiu highlighted the unique experiential advantage that her queer identity grants her as a translator, a benefit that outsider translators may find difficult to replicate: *“As a lesbian, I get the subtle cues in queer materials that outsider translators miss. The emotional nuances, the community slang, the unspoken feelings only someone who ‘understands the ins and outs’ can translate it authentically.”* She valued queer community approval and recognition far above any financial or professional reward: *“People in the local queer community say my translations ‘feel real’; that’s more important than any royalty check or publisher praise.”* She shared a specific small but meaningful translational choice that reflects this commitment to authenticity: *“For a northern Chinese lesbian couple novel, I translated the English ‘partner’ as ‘laopo’, Mandarin colloquial for wife, and the term our local queer community uses. This small detail makes the story authentic and relatable, mirroring our daily language.”*

Qing described queer translation as a dual practice of personal self-exploration and queer community solidarity, framing his work as a bridge between different queer experiences: *“I’m gay, and translating queer works helps me explore new perspectives beyond my own small circle. It’s a meaningful collision of ideas, and it lets me share our diverse queer culture with both other queer people and mainstream readers.”* Despite facing social resistance and misunderstanding from friends and family, he remained resolute in his advocacy: *“Some friends around me question my focus on these books, but their doubt fuels me. I believe translation changes minds and builds understanding, one book at a time.”* He explained how he balances strict authenticity for queer insiders with accessibility for non-queer readers: *“I keep queer slang and community terms intact but add brief, unobtrusive explanations in parentheses for non-queer readers; this way, insiders feel that I can ‘capture the essence’, and outsiders can learn without feeling alienated or overwhelmed.”*

Additional participants, including Kun, Lan, Yang, and Dan, reinforce this pattern by emphasizing emotional fidelity, intersectional inclusion, community empowerment, and resistance to erasure as central translational commitments. Kun prioritized emotional fidelity to queer lived experience for local regional audiences, rejecting the watered-down translations that are common in mainstream publishing: *“queer readers from the Yangtze River Delta need authentic translations, not publisher-sanitized versions. I prioritize emotional fidelity to mirror our real joys, pains, hopes, and frustrations.”* Lan introduced a critical intersectional awareness to her queer translation practice, pushing back against the erasure of bisexual identities in both mainstream and queer discourse: *“As a bisexual woman, I translate queer content to highlight the diversity of our community. It’s not just about gay or lesbian stories, we are a spectrum of identities, and that diversity needs to come through in the translations.”*

Yang sees his translation as community empowerment from an insider view: *“I understand the struggle, hidden joys, and longing for recognition.”* His work makes queer readers feel seen and less alone. He preserves insider terms for authenticity while keeping mainstream readers oriented, viewing translation as a bridge and affirmation. Dan calls her work quiet resistance against erasure: *“Translating queer narratives is resistance. Our stories deserve authentic telling.”* She refused to soften a queer character’s background for a publisher, losing the project but upholding integrity, making her work relatable to local queer communities.

Thematic analysis of these accounts points to a distinct, identity-centered habitus among many of these insider advocates. Across participants’ narratives, repeated emphasis on emotional truth, cultural specificity, queer community responsibility, community empowerment, and resistance to erasure supports this emerging theme, which can contribute to challenging the dominant pattern of erasure and marginalization. By drawing on their insider cultural capital, lived queer experience, knowledge of regional queer slang and norms, and regional queer social networks, these participants produce translations that often support queer identity affirmation, cultural preservation, and low-key community advocacy within structurally constrained environments. Their accounts illustrate how queer translators may use their unique positional advantage to create texts that resonate with marginalized queer audiences, capturing subtle emotions and lived experiences that outsider translators may find difficult to render, even as they navigate the external pressures of censorship and mainstream publishing norms. For these participants, the ethical burden of translation lies not only in negotiating institutional constraint but also in representing queer communities without surrendering cultural specificity to sanitizing or market pressures.

### Translation as allyship: activist translation practices beyond identity

Six participants who identified as non-queer functioned as allied activists, whose engagement with queer translation is driven by a strong sense of ethical solidarity and a deep commitment to social justice and queer equality. Their accounts are characterized by moral reasoning, genuine empathic language, and concrete references to regional experiences of queer discrimination and marginalization, reflecting an ethics-driven habitus and a deliberate deployment of their cultural, linguistic, and social capital for queer advocacy. Unlike cautious negotiators, they openly acknowledge and embrace the political dimensions of their translation work, framing translation as a tangible tool for challenging injustice, expanding queer visibility, and building cross-identity inclusion.

Bo was motivated by his firsthand observation of the structural barriers to queer knowledge and information access in his region: *“A gay friend struggles to access queer books online, which infuriates me. Knowledge is not a privilege of sexuality, so I translate these texts for regional queer people to read freely.”* He framed his queer translation work in universal ethical terms, rejecting the idea that queer literature is only for queer audiences: *“These stories deserve to be read by everyone, not just the queer community. It’s a basic question of justice and equal access to culture.”* He added specific details about his grassroots outreach efforts that extend far beyond commercial publishing: *“I share my translations on underground queer literary platforms and send free printed copies to queer community centers in local cities. Money is not the point at all; access and visibility are.”* His motivation stems from, in his words, *“a desire to ‘defend against injustice’ and ensure equal access to queer narratives.”*

Ying connected her queer translation practice to her feminist identity and personal witnessing of queer suffering, drawing a direct link between patriarchal oppression and queer marginalization: *“A lesbian friend was forced into a loveless heterosexual marriage by her family, watching her suffer every day haunted me. I translate queer texts to amplify their voices and challenge the patriarchal norms that hurt queer people.”* She pursued a visceral affective impact in her translations, aiming to build empathy among straight mainstream readers: *“I want readers to feel what queer people feel, not abstractly or intellectually, but viscerally. Empathy is the first step to real change and acceptance.”* She explained the specific stylistic choices she makes to achieve this emotional resonance: *“I use vivid sensory language for emotional queer scenes instead of bland phrasing, making the love, loss, joy, and pain tangible for straight readers without such experiences.”*

Mei focused on addressing the unique isolation of queer people in Western China, where queer resources and community are far scarcer than in coastal cities:

*Queer people in Western China face a unique kind of isolation, especially in smaller cities and rural areas where there are no queer community centers or support networks. My translations aim to bridge that gap, lending a hand to queer people in isolated regions, providing them with a sense of connection and belonging.*

Ling expressed a strong, unshakable moral obligation that drives her queer translation work, framing it as a basic act of human decency: *“I’m not queer, but I cannot stand injustice and discrimination. Translating these queer texts is my way of standing with marginalized groups and challenging the heteronormativity that pervades our culture.”* Xiao emphasized linguistic and cultural accessibility as the core goal of his queer translation work, addressing the language barrier that excludes many Chinese queer people from global queer culture: *“Most key meaningful queer texts are only in English, so translating them into Chinese is an act of inclusion. I aim to make these stories accessible to non-English speakers in remote areas and small cities with no other queer culture access.”* Yue further framed queer translation as a practice of amplifying marginalized voices, highlighting its role in making underrepresented perspectives more visible within constrained cultural environments.

Thematic analysis suggests that this group often frames queer translation through ethical vocabulary, genuine empathic description, and justice-oriented reasoning as a form of ethical solidarity. In these accounts, participants leverage their professional linguistic expertise, access to mainstream publishing and cultural channels, and relative social privilege as non-queer people to expand access to queer narratives and foster cross-identity empathy, framing translation as a legitimate and meaningful form of allyship. In doing so, their actions can contribute to creating more inclusive spaces of representation for queer people in China and may challenge heteronormative structures from outside the queer identity group itself, without appropriating or centering their own experiences.

These accounts indicate an ethics-driven orientation in which translation is understood not merely as professional work, but as a form of solidaristic cultural mediation. They illustrate one way in which non-queer translators may act as allies by centering marginalized queer voices, using their positional advantage to navigate structural constraints and amplify queer stories and identities. At the same time, these accounts point to an important ethical tension: allyship in translation requires supporting queer visibility without re-centering non-queer authority, paternalism, or symbolic ownership over the stories being translated.

## Discussion

Translation is inherently ideological and sociological (Bachleitner, 2019). In China, the translation of queer materials unfolds within a stratified and regionally diverse field shaped by state surveillance, heteronormative norms, market forces, and varying regional constraints (Chen, 2010). Historically, translation has played an important role in state-building projects in China, from the transmission of Buddhist texts to the importation of Marxist ideology (Sun, 2025). This historical legacy, together with contemporary sociopolitical forces, contributes to the formation of translators’ habitus and influences how translators recalibrate their professional choices and translation strategies in response to shifting expectations and constraints within the field (Chen, 2010). Against this backdrop, queer translation is not merely a matter of textual transfer, but a socially contested practice through which visibility, legitimacy, and cultural meaning are selectively negotiated under unequal conditions of power.

The 23 translators in this study function as socially situated agents operating within a field where economic, cultural, symbolic, and ethical forms of capital are unevenly distributed and strategically mobilized. Their practices illustrate how translation decisions are shaped by habitus, understood as internalized dispositions formed through education, identity, professional experience, and social positioning (Bourdieu, 1992). The four positions identified in this study, namely visibility-seekers, cautious negotiators, insider advocates, and allied activists, reflect distinct configurations of motivation and capital mobilization. These positions demonstrate how translators actively negotiate sociopolitical pressures while shaping the visibility and circulation of queer narratives. More specifically, visibility-seekers mainly pursue economic and symbolic capital in commercial fields; cautious negotiators rely on institutionally acceptable cultural capital within censored publishing contexts; insider advocates draw on insider cultural and social capital rooted in queer lived experience and community networks; and allied activists mobilize professional and social resources to support visibility across identity boundaries.

Visibility-seekers exhibit a market-oriented habitus that emphasizes the accumulation of symbolic and economic capital through the translation of queer materials. Their practices align with Bourdieu’s (1987) concept of instrumentalized cultural capital and correspond to the ‘Gay Warrior’ archetype identified by Cloud (2018), in which marginalized identities are framed in ways that resonate with dominant institutional values. In China, this often involves presenting queer content as entertainment that responds to consumer demand, including the popularity of CP fandom culture among young audiences (Zhou et al., 2022). Such strategies commodify queer identities as cultural currency, reflecting Venuti’s (2002) critique of depoliticized difference. Familiarity with fandom discourse and audience preferences operates as commercially usable cultural capital that may be converted into visibility, reputation, and economic return. Thematic patterns suggest that this market-driven orientation raises ethical questions regarding the influence of commercial incentives on translator agency and the representation of queer identities (Cui, 2023).

Cautious negotiators adopt a risk-averse habitus shaped by institutional constraints and censorship. Their strategies reflect what Inghilleri (2005) describes as managed agency, in which translators balance professional commitment with the need to navigate regulatory systems. Findings show that participants often employ pragmatic compromises such as adopting scholarly neutrality (Zhou and Liu, 2023), softening explicit language (Shaw and Zhang, 2017), or using euphemism and strategic vagueness to maintain the circulation of queer narratives while minimizing political risk. These recurring practices indicate a preference for professionally sustainable engagement rather than explicit representational affirmation. Censorship functions not only as an external constraint but also as a structuring force that shapes translators’ professional self-understanding, acceptable vocabularies, and perception of what can be publicly expressed. While such strategies support a limited form of queer visibility, they may also reproduce dominant heteronormative ideologies, resonating with Simon’s (1996) critique of neutrality as complicity.

Insider advocates reflect an identity-centered habitus in which translation is closely tied to cultural authenticity, emotional truth, and community affirmation. Their engagement suggests that lived queer experience can function as insider cultural and social capital, which Tian et al. (2025b) identify as important for resisting the stigmatization and misrepresentation of rainbow communities in Chinese translation contexts. By contrast, translators without such insider ties may encounter identity dilemmas and self-censorship (Tian and Yuan, 2025), often shaped by heteronormative pressures and institutional constraints associated with reduced translator agency (Tian et al., 2025a). Thematic patterns indicate that insider advocates draw on their community embeddedness to prioritize representational accuracy, affective resonance, and community responsibility, enabling them to avoid reductive portrayals and sustain marginalized meanings. For these participants, translation functions not only as professional work but also as a means of cultural preservation and subtle advocacy under constraint, aligning with the positive agency framework outlined by Tian et al. (2025a) and responding to the multi-layered stigmatization documented by Tian and Yuan (2025). In this sense, identity-informed translation may serve as a form of resistance to the identity tensions and professional dilemmas identified by Tian et al. (2025b).

Allied activists demonstrate an ethics-informed habitus that frames translation as a form of cross-identity solidarity. Their engagement reflects Bourdieu’s (1987) notion of disinterested investment, prioritizing ethical commitment and social justice over personal recognition or economic gain. Consistent with the ‘Gay Victim’ archetype identified by Cloud (2018), their translations draw attention to structural injustices affecting queer communities. Thematic patterns suggest that allyship involves prioritizing emotional truth, accessibility, and affective engagement (Karnaze et al., 2023), supporting Cvetkovich’s (2003) argument that affect can function as a politically generative force. Participants’ accounts indicate that cross-identity solidarity requires careful negotiation to amplify queer voices without re-centering non-queer authority, paternalism, or symbolic ownership. The findings point to allyship as both a moral orientation and a practical strategy that must navigate ethical tensions while promoting inclusive representation.

Regionality appears in this study as a noteworthy but preliminary cross-cutting pattern rather than a fixed explanatory dimension. Translators located in Northern and Central regions in this sample tended to report stricter censorship and therefore adopt more cautious strategies, while translators in Southern and Western regions more frequently described relatively flexible room for strategic experimentation. The comparatively stricter orientation reported by participants in Northern and Central regions may be understood in relation to their proximity to political and cultural centers. These regions are more closely embedded in national-level regulatory and institutional networks, where publishing practices are subject to tighter oversight and more formalized review procedures. In such contexts, both institutions and individual translators may exhibit heightened sensitivity to policy boundaries and potential risks, leading to more cautious and conservative translational strategies.

By contrast, in the case of Western China, the apparent flexibility can be better understood in relation to specific structural conditions rather than as an indication of greater cultural permissiveness. Compared with the more developed coastal regions, where foreign trade is active and translation demand is high, the inland translation market in Western China remains relatively small in scale and less institutionalized (China Translators Association, 2025). As a result, regulatory oversight tends to be looser and industry standards less standardized, which in turn allows greater discretion in translators’ actual practices and creates more room for flexible handling of source texts. At the same time, this flexibility is also shaped by market competition under conditions of limited demand. Despite the relatively small market size, the number of translators remains considerable, meaning that practitioners must compete for scarce opportunities. Under such circumstances, some translators adopt more adaptive and opportunistic strategies in order to secure visibility and access to projects. This may include a greater willingness to engage with diverse or unconventional genres, including works related to sexual minorities, which can sometimes attract attention and help consolidate one’s position within the market.

Across these positions and regional contexts, translators function as active agents embedded within power-laden structures. Their translation practices reveal continuous negotiation between conformity and resistance, whether through market alignment, cautious adaptation, insider advocacy, or ethical allyship. In this sense, translation becomes a site where competing ideological, economic, and ethical forces intersect, shaping both translation strategies and the public visibility of queer narratives (Spivak, 2021; Venuti, 2017).

## Conclusion

This study investigated what motivates 23 professional translators across China’s five major regions to engage with queer materials and how they navigate sociopolitical, cultural, and regional constraints. Drawing on Bourdieu’s framework, it identifies four distinct positions, including visibility-seekers, cautious negotiators, insider advocates, and allied activists, shaped by habitus, capital mobilization, and regional field conditions. The findings show that translators’ motivations range from professional ambition and market responsiveness to identity-informed advocacy and ethical solidarity. Despite these differences, participants actively engage with queer materials, suggesting that translation functions as a site of ongoing ideological negotiation in Chinese queer contexts.

This study contributes to queer translation studies by centering the Chinese regional context and providing an empirical corpus of professional translator perspectives. It also advances translation studies by demonstrating how a sociologically informed thematic analysis enables the identification of recurring patterns in translator positioning and reveals how different forms of agency emerge under ideological constraint, while also highlighting the role of local field conditions in shaping translational practices. The study underscores the translator’s role as an active cultural mediator, showing how translators negotiate legitimacy, social meaning, and inclusion through their engagement with queer materials, and how translation can function as a site of subtle activism within structurally constrained contexts.

A key limitation concerns the interpretation of regional variation. Although the sample is geographically diverse, its size does not support strong regional generalization, and the regional patterns identified here should therefore be understood as exploratory rather than definitive. This is especially important in relation to Western China, where the limited evidence in this study suggests not a uniformly more permissive environment, but a context in which low-visibility and community-oriented practices may give rise to different forms of strategic flexibility under conditions of resource scarcity. Future research could expand the sample to include grassroots translators and queer community members, conduct comparative thematic analyses of translated texts as well as translator and reader responses, and explore how digital platforms shape regional queer translation practices.

## Data Availability

The original contributions presented in the study are included in the article/supplementary material, further inquiries can be directed to the corresponding author/s.
